# Effect of conventional milling on the nutritional value and antioxidant capacity of wheat types common in Ethiopia and a recovery attempt with bran supplementation in bread

**DOI:** 10.1002/fsn3.315

**Published:** 2015-11-19

**Authors:** Genet Gebremedhin Heshe, Gulelat Desse Haki, Ashagrie Zewdu Woldegiorgis, Habtamu Fekadu Gemede

**Affiliations:** ^1^ Center for Food Science and NutritionCollege of Natural SciencesAddis Ababa UniversityP.O. BOX 1176Addis AbabaEthiopia; ^2^ Department of Food Science and TechnologyBotswana College of AgriculturePrivate Bag 0027GaboroneBotswana; ^3^ Department of Food Technology and Process EngineeringWollega UniversityP.O. Box: 395NekemteEthiopia

**Keywords:** Antioxidant, bran, nutritional, refined milling, wheat, white flour

## Abstract

The effect of wheat flour refined milling on nutritional and antioxidant quality of hard and soft grown in Ethiopia was evaluated. Bread was prepared with the supplementation of the white wheat flour with different levels (0%, 10%, 20%, and 25%) of wheat bran. Whole (100% extraction) and white wheat (68% extraction) flours were analyzed for proximates, minerals, and antioxidants. Results indicated that at a low extraction rate (68%), the protein, fat, fiber, ash, iron, zinc, phosphorous, and antioxidant contents of the samples significantly (*P* < 0.05) decreased by milling. The TPC (total phenolic content) of the white wheat flours, which ranged from 3.34 to 3.49 mg GAE (gallic acid equivalent)/g, was significantly (*P* < 0.005) lower than those of the whole wheat flours, whose TPC ranged from 7.66 to 8.20 GAE/g). At 50 mg/mL, the DPPH (2‐diphenyl‐1‐picrylhydrazyl) scavenging effect of the wheat extracts decreased in the order of soft whole, hard whole, soft white, and hard white wheat flour, which was 90.39, 89.89, 75.80, and 57.57%, respectively. Moreover, the proximate and mineral contents of the bran‐supplemented breads increased significantly (*P* < 0.05) with the bran level of the bread, and the highest values (protein, 12.0 g/100 g; fat, 2.6 g/100 g; fiber, 2.5 g/100 g; ash, 3.3 g/100 g; iron, 4.8 mg/100 g and zinc, 2.33 mg/100 g) were found in 25% bran supplemented bread. The sensory evaluation of bread showed that all the supplementation levels had a mean score above 4 for all preferences on a 7‐ point hedonic scale. The results indicated that refined milling at 68% extraction significantly reduces the nutritional and antioxidant activity of the wheat flours. Bread of good nutritional and sensory qualities can be produced from 10% and 20% bran supplementations.

## Introduction

Wheat has accompanied humans since remote times (as far back as 3000–4000 BC) in their evolution and development, evolving itself (in part by nature and in part by manipulation) from its primitive forms (emmer wheat) into the presently cultivated species (Curtis et al. 2002).Wheat crop is widely adapted to a variety of environments and is cultivated in tropical, subtropical, and temperate areas (Hussain et al. 2010). It is widely consumed by humans in over 100 countries that are primary producers and in other countries where wheat cannot be grown (Shewry 2009). It also occupies 27% of the total cereal production worldwide (Curtis et al. 2002). It is thus, an important agricultural commodity, which is consumed in large amount all over the world among all grains.

Ethiopia is the largest wheat producer in sub‐Saharan Africa (MOA, 2011). Nationally, wheat ranks fourth in total area coverage (1,389,215.00 ha). It is also third in productivity (after maize and sorghum) among cereals (CSA, 2005). It is one of the most important crops grown and consumed in Ethiopia both in terms of total production (2.85 million MT in 2010/11) (CSA, 2011) and the proportion of total calories consumed in the country (19.6% of calories consumed) (Rashid et al. 2010).

Wheat possesses several health benefits, especially when utilized as a whole‐grain product. According to Kumar et al. (2011), wheat provides protection against diseases such as constipation, ischaemic, heart disease, diverticulum, appendicitis, diabetes, and obesity. These benefits are attributed in part to the presence of different compounds such as dietary fibers, phytochemicals, proteins, vitamins, and minerals (Ragaee et al. 2012). Whole‐wheat grain consists of bran, germ, and endosperm. When conventionally milled, only carbohydrate‐ rich endosperm is retained. This results in a big loss of many nutritionally valuable biochemical compounds such as dietary fiber, vitamins, minerals, and antioxidant compounds, which play an important role in reducing CVD (cardiovascular disease) (Mellen et al. 2008). When white flour is produced, many important nutrients and fiber are removed because these components are mainly located in bran and germ (Iuliana et al. 2012). Wheat bran is rich in protein (~14%), carbohydrates (~27%), minerals (~5%), and fat (~6%) (Anwarul et al. 2002). In addition, wheat bran is the main by‐product of conventional flour milling. Wheat bran is a most important fiber source, which is inexpensive and available. It is a good source of not only dietary fiber. The loss of vitamins and minerals in the refined wheat flour has led to widespread prevalence of constipation and other digestive disturbances and nutritional disorders (Kumar et al. 2011).

Milling is the critical process affecting the concentrations of nutrients in wheat‐derived food products. The outer parts of the kernel, especially the aleurone layer and the germ are richer in minerals. Conventional milling reduces nutritional content of flour and concentrates them in the milling residues (Cubadda et al. 2009). White flour with a milling extraction rate 68% mean up to 32% of the original grain is not in the flour. Whole grain flour includes all parts of the seed and is 100% milling extraction rate. Milling of wheat into highly refined flours not only precludes considerable amounts of nutrients from human consumption, but the remaining flours have a much poorer nutritive value than flours made from whole wheat.

Over the past 20 years, wheat production and consumption have both increased in Ethiopia (Bergh et al. 2012). In Ethiopia, 28% of consumers purchase wheat flours and flour products, and about 22 million people use wheat flours (FDRE, 2011). However, no published information is available regarding the effects of conventional milling refining on nutritional and antioxidant capacity of wheat that are commonly grown in Ethiopia, though wheat is widely distributed and consumed. The nutritional value and antioxidant properties of wheat grain are significantly influenced by soil type and richness, growing temperatures, moisture levels, other climatic differences, and genotype (Adom et al. 2003). It is therefore, very important to understand the nutritional value of Ethiopian wheat and evaluate the effects of conventional milling. In addition, it is necessary to find a way to improve the nutrient quality of wheat products without compromising palatability.

## Materials and Methods

### Samples

**Figure 1:**
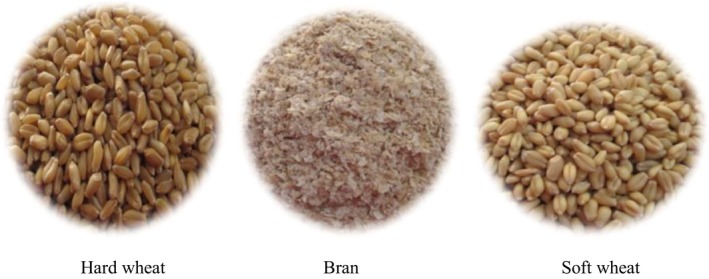
Hard wheat bran soft wheat.

Hard wheat (Kubsa) and soft wheat (ET‐13) samples were obtained from the Kebron food complex (Oromia region) and Wedera farmers cooperative (Debrebrhan), respectively, in Ethiopia. Bran sample was obtained from the Universal Food Complex (Addis Ababa, Ethiopia) (Fig. 1).

### Milling of wheat

The amount of water required for tempering was calculated according to AACC (2000). One kilogram of each sample from both the soft and hard wheat were cleaned and tempered separately to 14% moisture level and kept for 6 and 24 h, respectively, at ambient temperature in a closed plastic jar. After tempering, wheat samples were milled at the extraction rates of 68% and 100% by using. The milling of the flour was conducted at Kokeb Flour and Pasta Factory and extraction rate was calculated according to Slavin et al. (2000).

### Formulation of bread

Flour blends were prepared by mixing wheat flour with wheat bran in the proportions of 100: 0, 90:10, 80:20, and 75:25 (wheat flour to bran) using homogenizer and 100% white wheat flour was used as the control. The formulation was made based on the preliminary test (unpublished). The four flour samples were packaged in black low‐density polyethylene bags and stored in plastic containers at room temperature from where samples were taken for bread production.

### Bread manufacture

Bread was prepared with the formulated flours in 2.3, and the dough was prepared based on the method described by Hertzberg and Francois (2007) with some modifications; Each formulated flour (400 g) was mixed (Linkrich‐B15; with sugar (8 g), salt (4 g), oil (8 g), and yeast (1.6 g; baker' s yeast) for 30 min and then water was added to the dough for the desired consistency. The dough was weighed and divided into three equal portions for replications. These were placed in baking pans and left for 1 h. Then they were transferred into an oven preheated to about 180–250°C and allowed to bake for 20 min. The baked products were left to cool.

### Nutritional analysis of wheat flour, bran and bread

All samples were analyzed for moisture, crude protein, crude fat, and total ash by standard methods (AOAC, 2000).

### Determination of mineral contents

Iron and zinc were determined according to the standard method of AOAC (2000) using flame Atomic Absorption Spectrophotometer. Ash was obtained from dry ashing of the samples. The ash was wetted completely with 5 mL of 6N HCl, and dried on a low temperature on hot plate. A 7 mL of 3N HCl was added to the dried ash and heated on the hot plate until the solution just boiled. The ash solution was cooled to room temperature in a hood and was filtered using filter paper (Whatman 45). A 5 mL of 3N HCl was added into each crucible dishes and was heated until a solution boiled then cooled and filtered into the flask. The crucible dishes are again washed three times with deionized water, the washing was filtered into a flask. Then, the solution was cooled and diluted to 50 mL with deionized water. A blank was prepared by taking the same procedure as the sample.

#### Sample extraction

Samples were extracted based on the procedures as outlined by Woldegiorgis et al. (2014). The powdered wheat samples were homogenized and weighed (10 g) before extraction by stirring with 100 mL of methanol at 250 C at 150 rpm for 24 h using an incubator shaker (ZHWY‐103) and then filtered through Whatman No. 1 filter paper. The residue was then extracted with two additional 100 mL portions of methanol as described above. The combined methanolic extracts were evaporated at 400°C to dryness using a rotary evaporator and redissolved in methanol at a concentration of 50 mg/mL and stored at 40°C for further use.

#### Determination of free radical scavenging activity

The hydrogen atoms or electrons donation ability of the corresponding extracts and some pure compounds were measured from the bleaching of purple colored methanol solution of DPPH (Gursoy et al. 2010). Antioxidant activity of the methanol extracts was determined by DPPH radical scavenging method as described by Woldegiorgis et al. (2014). A 0.004% solution of DPPH radical solution in methanol was prepared and then 2 mL of DPPH solution was mixed with 1 mL of various concentrations (0.1–50 mg/mL) of the extracts in methanol. Finally, the samples were incubated for 30 min in the dark at room temperature. Scavenging capacity was read spectrophotometrically by monitoring the decrease in absorbance at 517 nm. Ascorbic acid was used as a standard and mixture without extract was used as the control. Inhibition of free radical DPPH in percent (I %) was then calculated.

#### Total phenolics determination

Phenolic compounds concentration in the wheat was estimated with Folin–Ciocalteu reagent according to the Singleton & Rossi method (1965) as described by Woldegiorgis et al. (2014). One milliliter of sample (5000 *μ*g) was mixed with 1 mL of Folin and Ciocalteu's phenol reagent. After 3 min, 1 mL of saturated sodium carbonate (20%) solution was added to the mixture and adjusted to 10 mL with distilled water. The reaction was kept in the dark for 90 min, after which the absorbance was read at 725 nm. Gallic acid was used to construct the standard curve (5–80 *μ*g/mL). The results were mean values + standard error of mean and expressed as mg of GAEs (gallic acid equivalents/g of extract).

### Sensory evaluation of bread

Sensory evaluation was conducted for the freshly baked breads by 30 semitrained panelists consisting of male and female students, aged from 23 to 43 years old, from the Food Science and Nutrition Center of the Addis Ababa University. The samples were presented randomly in identical containers, coded with three digit numbers. The sensory test was conducted using a seven‐ point hedonic scale, where 1 = dislike very much, 2 = dislike moderately, 3 = dislike slightly, 4 = neither like nor dislike, 5 = like slightly, 6 = like moderately and 7 = like very much. The sensory attributes evaluated were taste, odor, color, texture, and overall acceptability. Samples were considered as acceptable when their average score for the overall acceptability was >4 (neither like nor dislike) (Lazaridou et al. 2007).

### Statistical analysis

The data were subjected to ANOVA (analysis of variance) and Duncan's multiple range tests were used for mean separation at *P* < 0.05. Linear regression analysis was used to calculate IC50 value. Pearson correlation between DPPH scavenging (%) and TPC (total phenolic content) was considered at *P* < 0.05.

## Results and Discussion

### Proximate composition of wheat, wheat bran, and bread

The mean value for moisture, crude protein, crude fat, total ash and crude fiber of wheat bran, wheat flour (hard and soft), white wheat flour (hard and soft), and bran supplemented bread are presented in Tables 1, 2, 3. The mean values for moisture contents of different whole wheat and white wheat flours are presented in Table 1. It ranged from 10.5% to 12.3%. The highest moisture level, 12.3%, was found in hard white wheat flour. The moisture content varies significantly between whole and white flour and between hard and soft white flour (*P* < 0.05).The increment on moisture content of both soft and hard white wheat as compared to the whole wheat flour could be due to the addition of water during the tampering process to facilitate milling of wheat, which resulted in retaining more water in refined wheat flour than whole wheat flour.

**Table 1:** Proximate composition of wheat

Parameters	Wheat samples			
	HWF	HWWF (refined)	SWF	SWWF (refined)
Moisture (%)	10.75 ± 0.38^a^	12.30 ± 0.09^c^	10.48 ± 0.10^a^	11.60 ± 0.23^b^
Ash (%)	1.62 ± 0.03^d^	0.65 ± 0.01^b^	1.41 ± 0.07^c^	0.38 ± 0.07a
Fat (%)	1.82 ± 0.04^b^	1.43 ± 0.18^a^	1.78 ± 0.10^b^	1.32 ± 0.11^a^
Protein (%)	14.40 ± 0.30^d^	11.91 ± 0.087^c^	9.11 ± 0.12^b^	7.13 ± 0.06a
Fiber (%)	2.6 ± 0.08^b^	0.42 ± 0.06^a^	2.5 ± 0.08^b^	0.36 ± 0.07^a^

HWF, Hard whole wheat flour; HWWF, Hard white wheat flour (refined); SWF, Soft whole wheat flour; SWWF, Soft white wheat flour (refined).

Data are average of triplicate ± SE.

Mean value with different superscript in the same rows are significantly different (*P* < 0.05).

**Table 2:** Proximate composition of wheat bran

Parameter	Composition (g/100 g)
Protein	15.26 ± 0.35
Fat	3.12 ± 0.7
Fiber	9.97 ± 0.27
Ash	4.5 ± 0.16

**Table 3:** Proximate composition of Bran supplemented bread and control (Dry weight basis). WFB‐(white wheat flour bread) – control, WF:BR 90:10, 10% bran supplemented bread, WF:BR 80:20 – 20% bran supplemented bread, WF:BR 75:25 – 25% bran supplemented bread

Samples	Moisture %	Protein %	Fat %	Ash %	Fiber %
Control (WFB)	30.92 ± 0.38^a^	9.42 ± .22^a^	1.56 ± .0.06^a^	1.38 ± 0.10^a^	0.38 ± 0.02^a^
WF:BR (90:10)	33.14 ± 0.26^b^	10.70 ± 0.55^b^	2.14 ± 0.032^b^	2.04 ± 0.32^b^	2.13 ± 0.05^b^
WF:BR (80:20)	33.24 ± 0.69^b^	11.66 ± 0.89^c^	2.36 ± 0.04^c^	2.29 ± 0.04^c^	3.11 ± 0.06^c^
WF:BR (75:25)	32.83 ± 0.58^b^	12.04 ± 0.84^c^	2.61 ± 0.06^d^	2.48 ± 0.02^d^	3.27 ± 0.008^d^

Data are average of triplicate ± SE.

Mean value with different superscript in the same column are significantly different (*P* < 0.05).

There is also a significant difference (*P* < 0.05) on total ash contents of all flour samples (Table 1). The highest ash content (1.6%) was found in hard whole wheat flour whereas the soft white flour showed the lowest (0. 4%) ash content. The results were comparable to Azizi et al. (2006) values obtained from different extraction rate of wheat flour, which ranged 1.51% to 0.54% ash content at 93% and 70% extraction rate, respectively.

The result for crude fat content is shown in Table 1 and the values showed significant difference (*P* < 0.05) between whole wheat flours and white wheat flours. The fat content decreased in white wheat flour. The highest fat content, 1.83%, was found in whole wheat flour (100% extraction rate); whereas, the lowest, 1.32%, was found in white wheat flour (low extraction rate). The high percentage of fat in whole wheat flour is because wheat germ is ground along with endosperm during milling (Farooq et al. 2001).

The results of this study also indicated that the protein contents for all flours varied significantly (*P* < 0.05). The protein contents decreased with in both hard and soft white wheat flour; at the same time, there was significant difference between hard and soft whole wheat flour. The highest protein content found on hard whole wheat flour was 14.40%; whereas the protein content of soft whole wheat flour was 9.11%. The result of this study was in line with the value of hard and soft whole wheat reported by Blakeney et al. (2009).

The mean value for fiber content of hard whole wheat, hard white wheat, soft whole wheat, and soft white wheat flour are 2.6, 0.42, 2.5 and 0.36 g/100 g, respectively (Table 1). The result of this study showed that there is significant difference (*P* < 0.05) between whole wheat flour and white wheat flour. Both hard and soft whole wheat flour exhibited high crude fiber content (2.6 and 2.5%). The white wheat flour showed less fiber content because wheat bran was removed during milling process, which decreased the amount of fiber in flour. The result of this study is in agreement with Azizi et al. (2006), who reported crude fiber in the range of 0.30–2.24% white wheat and whole flour, respectively.

The proximate composition of wheat bran samples are given in Table 2. Wheat bran was found to contain highest amounts of crude protein, fat, fiber, and ash with mean values of 15.26%, 3.12%, 9.97%, and 4.45%, respectively (Table 2). The objective of milling is to separate the bran and germ from the starchy endosperm so that the endosperm can be ground into flour. The aleurone layer, which is rich in protein, minerals, and vitamins, usually breaks away with the outer layer of the bran in the milling process, thus, contributing significantly to the nutritional quality of the bran fraction (Posner 2000).

Proximate composition of different bran supplemented bread and control were also analyzed for proximate composition. The mean values for moisture contents of the bread samples are presented in Table 3, which ranged from 30.92% to 32.83%. The highest moisture level, 32.83%, was found in 25% bran supplemented bread. The moisture content of the control bread decreased from the three samples bread significantly (*P* < 0.05).

The statistical analysis for crude protein is presented in Table 3. The mean value for protein content of all the study samples of bread ranged from 9.42 for control to 12.04 for WBB (75:25). The protein contents for three of the breads (0%, 10%, 20% bran supplemented bread) varied significantly (*P* < 0.05). However, there was no significant difference between 20% and 25% bran supplemented bread. The result of protein contents are in agreement with the findings of Butt et al. (2004) who reported an increase in contents of protein with an increase in bran proportion.

The result of this study indicates that crude fat showed significant difference (*P* < 0.05) among all breads. The fat content increased with an increase in bran level. The highest fat content, 2.61%, is found in 25% bran supplemented bread, whereas the lowest, 1.56%, was found in the control bread. The increase in fat content is because of the germ which is grounded along with bran and endosperm during milling, results in bread with higher fat content than the control bread (Farooq et al. 2001).

The mean values of crude fiber contents of different bread samples are given in Table 3. The statistical analysis showed significant (*P* < 0.05) effect on the quantity of crude fiber. The crude fiber contents ranged from 0.38% to 3.27%. The 25% bran supplemented bread exhibited the highest crude fiber (3.27%), whereas the control bread contained the lowest crude fiber (0.38%). The crude fiber increased with an increase in bran supplementation rate. The control showed less fiber contents because the bread was made from refined bread with no addition of bran.

Ash is the mineral residue remaining after a sample has been completely oxidized in a manner such that all organic volatile material is driven off, while preventing any mineral from being lost (Posner 2000). Ash varied significantly among all the bread (Table 3). The statistical analysis showed significant (*P* < 0.05) effect on total ash contents. The results indicate that ash content ranged from 1.38% to 2.48%. The highest ash content (2.48%) was found in 25% bran supplemented bread, whereas the control showed the lowest (1.38%) ash content. The addition of 10% to 25% wheat bran to the bread increased the ash content.

### Mineral content of wheat flour and bread

The mineral content of the whole and white flour samples are shown in Table 4. According to the results of this study, the iron content level in hard and soft whole wheat flour is significantly different (*P* < 0.05) from hard and soft white wheat flour. The iron content of whole wheat flour ranged from 2.95 to 4.15 mg/100 g whereas the iron content of the white wheat flour ranged from 2.51 to 3.35 mg/100 g.

**Table 4:** Mineral composition of whole and refined wheat flour

Wheat samples	Mineral content mg/100 g		
	Iron	Zinc	Phosphorous
HWF	4.15 ± 0.12^c^	3.59 ± 0.063^d^	337.99 ± 0.56^d^
HWWF	2.51 ± 0.16^a^	1.39 ± 0.036^b^	144.69 ± 0.61^b^
SWF	3.35 ± 0.17^b^	2.47 ± 0.04^c^	313.98 ± 1^c^
SWWF	2.95 ± 0.26^a^	0.58 ± 0.01^a^	77.03 ± 0.51^a^

HWF, hard whole wheat flour; HWWF, Hard white wheat flour; SWF, soft whole wheat flour; SWWF, Soft white wheat flour.

Mean value with different superscript in the same column are significantly different (*P* < 0.05).

Dewettinck et al. (2008) reported that the iron content of the whole wheat was (1–5 mg/100 g), which is in agreement with the result of this study. However, the level of iron in the white flour decreases significantly. The milling process removes many important nutrients when white flour is produced. The bran and the germ are relatively rich in minerals and the milled products contain less of these than the original grain. As a result of milling, the palatability is increased, but the nutritional value of the products is decreased (Hoseney 1992).

Zinc content varied significantly among all whole wheat and white wheat flour (Table 4). The zinc content of the whole wheat ranged from 3.59 to 2.47 mg/100 g. However, the white flour contained zinc content in the range of 0.58 to 1.39 mg/100 g. The highest Zn content 3.59 mg/100 g was found in hard whole wheat flour. This study showed that the low rate of extraction of wheat reduce the zinc content of the wheat significantly (*P* < 0.05). The hard whole wheat flour zinc content was 3.59 mg/100 g whereas milling reduced the zinc content to 1.39 mg/100 g. Whereas, in case of soft wheat, the decrease was from whole wheat to white wheat flour 2.47 to 0.58 mg/100 g of zinc, respectively. According to Lopez et al. (2003), 80% of the total amounts of minerals are concentrated in the aleurone layer of pericarp (bran), which was removed during milling process while only 20% minerals are present in endosperm.

The mean total content of phosphorous on hard whole and soft whole wheat flour was 337.99 and 313.98 mg/100 g, respectively, however, there was significant decrease in phosphorous content (*P* < 0.05) on the refined milled product of hard and soft white wheat flour which was 144.69 and 77.03 mg/100 g, respectively. Wheat is one of the cereals which is classified as rich sources of phosphorous. The result of the whole wheat flour was in line with the finding of Dewettinck et al. (2008), which reported the presence of phosphorous from 200 to 1200 mg/100 g.

The replacement effect of different levels of wheat bran on the mineral content of bread is shown in Table 5. All the mean value for iron content varied among the bread samples. Results showed that the mineral progressively increased when levels of bran were increased. Control contained 1.98 mg/100 g iron and at the level of 25% bran replacement, the value increased to 4.84 mg/100 g of iron. The result of this study was in agreement with the study of Butt et al. (2004).

**Table 5:** Mineral analysis of bread. WFB‐(white wheat flour bread) – control, WF:BR 90:10, 10% bran supplemented bread, WF:BR 80:20 – 20% bran supplemented bread, WF:BR 75:25 – 25% bran supplemented bread

Samples	Iron mg/100 g	Zinc mg/100 g
Control (WFB)	1.98 ± 0.056^a^	0.93 ± 0.064^a^
WF:BR (90:10)	2.34 ± 0.159^b^	1.697 ± 0.108^b^
WF:BR (80:20)	2.95 ± 0.0753^c^	2.28 ± 0.131^c^
WF:BR (75:25)	4.83 ± 0.074^d^	2.33 ± 0.066^c^

Data are average of triplicate ± SE.

Mean value with different superscript in the same column are significantly different (*P* < 0.05).

The replacement effect of different levels of wheat bran on the zinc content of bread is shown in Table 5. Results showed that zinc progressively increased when levels of bran were increased. The result ranged from 0.93 to 2.33 mg/100 g of zinc. The value of zinc at the bran supplementation of between 20 and 25% was not significantly different (*P* > 0.05).

Micronutrient malnutrition greatly increases mortality and morbidity rates, diminishes cognitive abilities of children, lowers labor productivity, and reduces the quality of life for all those affected. Deficiency of micronutrients, such as iron and zinc, is critical and major problem. It could be concluded that the addition of bran improves the nutritional quality of bread and could be a means of providing adult their daily requirements of iron and zinc. Although supplementation of bran improves the mineral content of the bread due to increase in phytic acid content, the availability of these minerals may be reduced. Therefore, such issues would need further evaluation.

### Antioxidant capacity of wheat

The percentage yields of extracts were 7.58% w/w (Hard refined wheat), 8.2% w/w (Hard whole flour wheat), and 7.7% w/w (Soft refined wheat flour) and 7.3% w/w (soft whole flour wheat).

It has been recognized that the TPC of plant extracts is associated with their antioxidant activities due to their redox properties, which allow them to act as reducing agents, hydrogen donors, and singlet oxygen quenchers. TPC was expressed as milligrams of GAE (Gallic acid equivalent) per gram (mg/g) of dry flour samples. As shown in Table 6, the TPC in whole wheat was highest. The TPC of white wheat flours (refined), which ranged from 3.34 to 3.49 mg GAE/g which were significantly lower (*P* < 0.005) than those of whole wheat flours (range 7.66–8.20 mg GAE/g). However, the mean content did not vary much between whole hard and soft wheat type. Also, there was no significant variation between soft and hard white wheat flour. The difference in the TPC between whole and white wheat flour could be due to the process of milling. Research found antioxidants in wheat concentrated mostly in the aleurone layer of bran with some in the pericarp, nucellar envelope, and germ (Fulcher and Duke 2002; Žilic et al. 2012).

**Table 6:** Percent yield and total phenolics content

Sample	Yield % (g/100 g)	Total phenolics (mg GAE/g)
HWF (whole)	8.2	7.66 ± 0.70^b^
HWWF(refined)	7.58	3.49 ± 0.86^a^
SWWF (refined)	7.7	3.34 ± 0.14^a^
SWF(whole)	7.3	8.20 ± 0.35^b^

HWF, hard whole wheat flour; HWWF, Hard white wheat flour (refined); SWF, Soft whole wheat flour; SWWF, Soft white wheat flour (refined).

Data are average of triplicate ± SE.

Values in the same column with different superscript are statistically significant (*P* < 0.05).

The ability of wheat extracts to quench reactive species by hydrogen donation was measured through the DPPH radical scavenging activity test. The antioxidants can react with DPPH, a violet colored stable free radical, converting it into a yellow colored *α*,*α*‐diphenil‐*β*‐ picrylhydrazine. The discoloration of the reaction mixture can be quantified by measuring the absorbance at 517 nm, which indicates the radical scavenging ability of the antioxidant. The antioxidant capacity of whole and refined wheat was measured as the DPPH• scavenging activity.

**Figure 2:**
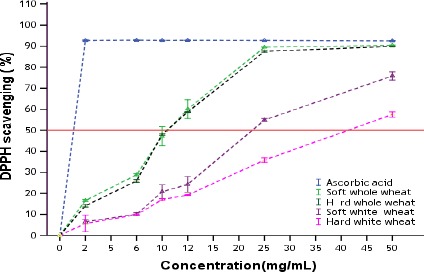
DPPH radical scavenging activity (%) of wheat extracts and standard (Values are average of triplicate measurements [mean ± SEM]).

The DPPH radical scavenging effects of wheat methanol extracts was shown in Figure 2. As the concentration of sample increased, the percent inhibition of DPPH radical also increased (Haung et al. 2005). At the concentration of 50 mg/mL, the scavenging effect of ascorbic acid, and wheat extracts, on the DPPH radical scavenging decreased in the order of L‐ ascorbic acid > soft whole > hard whole > soft white > hard white wheat flour, which were 92.53, 90.39, 89.89, 75.80, 57.57%, respectively. Therefore, the percentage of DPPH radical scavenging capacity of soft whole and hard whole wheat extracts are comparable with commercial antioxidants, L‐ ascorbic acid at concentration of 50 mg/mL. This suggested that whole wheat contain compounds that can donate electron/hydrogen easily and stabilizes free radicals.

The IC50 values of all the extracts were calculated from plotted graph of percentage scavenging activity against concentration of the extracts (Fig. 2). The lower the IC50 value, the higher is the scavenging potential. The IC50 values ranged from 10.56 mg/mL for whole wheat extracts to 41.25 mg/mL for hard white wheat extracts. Strongest scavenging activity (lower IC50 values) was recorded for whole hard and soft wheat extracts, which appeared more than four times stronger than that of hard white flour and two times stronger than that of soft white wheat extracts. IC50 value of ascorbic acid, a well‐known antioxidant, was relatively more pronounced than that of the extracts (Fig. 2). The results of this study demonstrate that the antioxidant content of wheat has been affected by the refined extraction/milling process. According to Fikreyesus et al. (2013), the DPPH (IC50) for whole wheat flour is 15.56, which is different form the result obtained in this research. It is known that the antioxidant properties of wheat grain are significantly influenced by the genotype and environmental conditions (Adom et al. 2003).To the best of our knowledge, there are no or few studies conducted on antioxidant content of Ethiopian wheat and particularly on comparison of antioxidant content on the whole and white wheat flour.

A relationship between phenolic content and antioxidant activity was extensively investigated, and both positive and negative correlations were reported. Bakchiche et al. (2013), Petra et al. (2012) and many other research groups stated that there was a positive correlation. However, a few evidences of no significant correlation were reported (Mohammad et al. 2008). In this study, the dependence of DPPH scavenging activity (%) in relation to the TPC was also evaluated. The TPC correlated significantly with DPPH scavenging activity (*R* ^2^ = 0.637, *P* < 0.05). Thus, the phenolics from the wheat extracts showed a good hydrogen‐donating capacity, as well as high reactivity to free radicals, leading to the stabilization and termination of the radical chain reactions.

### Sensory analysis of the bread

**Figure 3:**
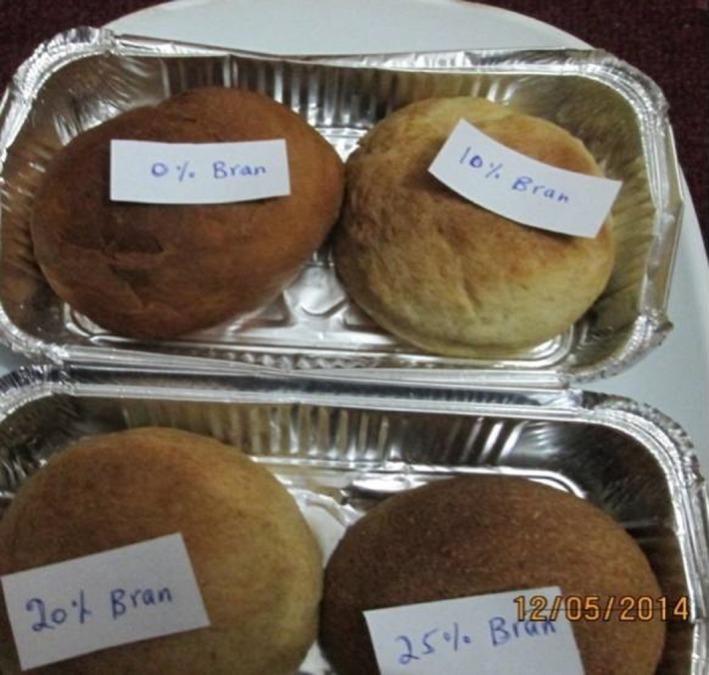
Wheat bran supplemented bread.

The sensory attributes of bread made from bran (Fig. 3) using different ratio were evaluated using 7‐point hedonic scale at Addis Ababa University; Center for Food Science and Nutrition by semitrained panelists of first year M.Sc program students of Food Science and Nutrition stream and the mean scores of evaluated sensory attributes were presented in Table 7.

**Table 7:** Sensory characteristics of wheat bran supplemented Bread. WFB‐(white wheat flour bread) – control, WF:BR 90:10, 10% bran supplemented bread, WF:BR 80:20 – 20% bran supplemented bread, WF:BR 75:25 – 25% bran supplemented bread

Sample	Test	Odor	Color	Texture	Overall acceptability
Control (WFB)	5.93 ± 0.14^c^	5.53 ± 0.21^b^	5.93 ± 0.18^c^	5.70 ± 0.19^b^	5.93 ± 0.11^c^
WF:BR (90:10)	5.26 ± 0.16^b^	5.43 ± 0.18^b^	5.46 ± 0.14^c^	5.30 ± 0.13^b^	5.43 ± 0.15^b^
WF:BR (80:20)	4.80 ± 0.24^b^	5.33 ± 0.23^b^	4.76 ± 0.22^b^	4.60 ± 0.20^a^	4.96 ± 0.16^b^
WF:BR (75:25)	4.43 ± 0.30^a^	4.76 ± 0.22^a^	4.13 ± 0.24^a^	4.36 ± 0.23^a^	4.50 ± 0.22^a^

Data are average of triplicate ± SE.

Mean value with different superscript in the same column are significantly different (*P* < 0.05).

Taste is an important parameter when evaluating sensory attribute of food. The product without acceptable good test is likely to be unacceptable by consumers. The observed mean score of taste in experiential bran supplemented bread ranged from 4.43 to 5.93 (Table 7). Control (100% white wheat flour bread) had the highest mean sore in taste (5.93) followed by 10% bran supplemented bread (5.93). The 100% white wheat flour (control) bread had significant difference (*P* < 0.05) with 10% bran (5.26), 20% bran bread (4.80), and 25% bran bread (4.43).

The control bread had significant difference (*P* < 5) from three of the bran supplemented bread (10%, 20%, 25%). The 10% bran supplemented bread scores >5 indicating that it is moderately likable by panelists, and 20% and 25% bran supplemented bread were rated as neither like nor dislike by the panelists (scored as 4.80 and 4.43, respectively). This is due to the addition of the bran to the bread.

The mean score of odor of bread ranged from 4.76 to 5.53 (Table 7). Most of the samples were similar in odor while only 25% supplemented bread significantly (*P* < 0.05) decreased. Three of the samples were liked moderately while 25% bran supplemented bread was rated as neither like nor dislike. As is shown in Table 7 color of bread had low score as a result of increasing the level of wheat bran. The color of control and 10% supplemented bread were similar in appearance while 20% and 25% bran supplemented bread were decreased (4.76 and 4.13) significantly (*P* < 0.05). The results indicated that no significant difference (*P* > 0.05) was observed by panelists between the control and 10% supplemented bread. The texture of the control and 10% bran supplemented bread were relatively most preferred (liked moderately) by the panelists. While the bread prepared from increasing level of bran supplement from 25% were scored as 4.5.

Generally, among the bread products, the control was highly acceptable by the panelists, with a score of 5.93. Next to this, 10% and 20% bran supplemented bread scored, 5.43 and 4.96, respectively. These two are liked moderately. The result obtained for 25% bran supplemented bread was significantly different from the previous two. The latter was neither like nor disliked by the panelists and it scored 4.5. In relation to this, Lazaridou et al. (2007) reported that those samples were considered as acceptable, which their average score for the overall acceptability were greater than 4 which mean neither like nor dislike. Thus, whole wheat flour with high extraction rate (100%) needed to be given high emphasis by consumers because of its nutritional and antioxidant capacity of the product.

## Conclusions

Wheat and wheat products are important staple foods that are commonly consumed in Ethiopia. Consumption of whole grains as part of the diet is recommended for health reasons because they are good source of minerals, fibers, protein, and antioxidants. There are no studies on the effect of refining on the nutritional content and antioxidant capacity of wheat grown in Ethiopia. This study showed that wheat extraction/refining at the lower rate have significantly reduced the proximate composition as well as the antioxidant content of wheat in both hard and soft wheat samples.

Addition of wheat bran to white wheat flour improves the nutritional value of the bread. Based on obtained results, the incorporation of wheat bran in the ratio of 10–20% showed better sensory acceptability though the proximate composition and mineral content increased at 25% bran supplemented bread. This indicates that bread of good nutritional and sensory qualities could be produced from 10% and 20% bran supplementation. The result of this study also indicated that wheat bran as a good source of minerals and fibers and can be used to supplement bread.

## Conflict of Interest

None declared.
