# Erratum

**DOI:** 10.1002/fsn3.460

**Published:** 2017-03-01

## Editorial Notice

It was recently brought to the attention of the editorial office that versions of the below articles published in *Food Science & Nutrition* were also published in the *Journal of Food Processing & Technology* (doi:10.4172/2157-7110.1000506) and the *Journal of Nutrition & Food Sciences* (doi:10.4172/2155-9600.S3-003).

Heshe, G. G., Haki, G. D., Woldegiorgis, A. Z. and Gemede, H. F. (2016), Effect of conventional milling on the nutritional value and antioxidant capacity of wheat types common in Ethiopia and a recovery attempt with bran supplementation in bread. *Food Science & Nutrition*, 4: 534–543. doi:10.1002/fsn3.315

Gemede, H. F., Haki, G. D., Beyene, F., Woldegiorgis, A. Z. and Rakshit, S. K. (2016), Proximate, mineral, and antinutrient compositions of indigenous Okra (*Abelmoschus esculentus*) pod accessions: implications for mineral bioavailability. *Food Science & Nutrition*, 4: 223–233. doi:10.1002/fsn3.282

Our editorial office investigated the situation and learned that the *Journal of Food Processing & Technology* and the *Journal of Nutrition & Food Sciences* published these articles without the author's consent and without the signature of a Copyright Agreement between the author and the Publisher. As such, we believe the articles published in *Food Science & Nutrition* should be the official published version of the articles.

